# ﻿*Pseudocryphonectriaelaeocarpicola* gen. et sp. nov. (Cryphonectriaceae, Diaporthales) causing stem blight of *Elaeocarpus* spp. in China

**DOI:** 10.3897/mycokeys.91.86693

**Published:** 2022-07-15

**Authors:** Hua-Yi Huang, Huan-Hua Huang, Dan-Yang Zhao, Ti-Jiang Shan, Li-Li Hu

**Affiliations:** 1 Guangdong Provincial Key Laboratory of Silviculture, Protection and Utilization, Guangdong Academy of Forestry, Guangzhou 510520, China Protection and Utilization, Guangdong Academy of Forestry Guangzhou China; 2 Guangdong Province Key Laboratory of Microbial Signals and Disease Control, College of Forestry and Landscape Architecture, South China Agricultural University, Guangzhou 510642, China South China Agricultural University Guangzhou China

**Keywords:** *Ascomycota*, phylogeny, plant disease, taxonomy

## Abstract

Cryphonectriaceae is a diaporthalean family containing important plant pathogens of which *Cryphonectriaparasitica* is the most notorious one. An emerging stem blight disease on *Elaeocarpusapiculatus* (Elaeocarpaceae) and *E.hainanensis* was observed in Guangdong Province of China recently. Typical Cryphonectria blight-like symptoms including cankers on tree barks with obvious orange conidial tendrils were observed. Forty-eight isolates were obtained from diseased tissues and conidiomata formed on the hosts *E.apiculatus* and *E.hainanensis*. These isolates were further identified based on both morphology and molecular methods using the combined sequence data of the internal transcribed spacer (ITS) region, large subunit of the nrDNA (LSU), the translation elongation factor 1-alpha (*tef1*) and DNA-directed RNA polymerase II second largest subunit (rpb2) genes. As a result, the fungus represents an undescribed genus and species within the family Cryphonectriaceae. Hence, *Pseudocryphonectriaelaeocarpicola***gen. et sp. nov.** is proposed herein to represent these isolates from diseased barks of *E.apiculatus* and *E.hainanensis*. *Pseudocryphonectria* differs from the other genera of Cryphonectriaceae in having dimorphic conidia. Further inoculation results showed that *P.elaeocarpicola* is the causal agent of this emerging blight disease in China, which can quickly infect and kill the hosts *E.apiculatus* and *E.hainanensis*.

## ﻿Introduction

Diaporthales represents a species-rich fungal order usually inhabiting plant tissues as pathogens, endophytes and saprophytes (Rossman et al. 2007; Senanayake et al. 2017; Voglmayr et al. 2017; Fan et al. 2018; Jaklitsch and Voglmayr 2020; Jiang et al. 2021a). Cryphonectriaceae is a pathogenic family within Diaporthales including several serious plant pathogens (Gryzenhout et al. 2006; Chen et al. 2016). For example, *Cryphonectriaparasitica* causes chestnut (*Castanea* spp.) blight disease worldwide (Rigling and Prospero 2017); *Chrysoportheaustroafricana*, *Ch.cubensis* and *Ch.deuterocubensis* result in eucalypt (*Eucalyptus* spp.) canker diseases in Africa, South America and Asia, respectively (Ferreira and Henfling 1976; Wingfield 1989; Old et al. 2003; Wang et al. 2020).

In a recent study, the family Cryphonectriaceae was re-evaluated based on morphological and molecular data of the ex-type strains, which accepted two subclades in the family with 21 genera and 55 species (Jiang et al. 2020). Subsequently, *Capillaureum* and *Parvosmorbus* were added to this family evidenced by both morphology and phylogeny (Ferreira et al. 2019; Wang et al. 2020). Currently, 23 genera were classified in Cryphonectriaceae based on morphological characters and combined sequence data of the internal transcribed spacer (ITS) region, large subunit of the nrDNA (LSU), and the translation elongation factor 1-alpha (tef1) and DNA-directed RNA polymerase II second largest subunit (*rpb2*) genes (Wijayawardene et al. 2018; Hyde et al. 2020; Jiang et al. 2020; Wang et al. 2020).

Cryphonectriaceae members are characterized by typical diaporthalean characters of perithecia with elongate beaks, often forming within stromatic tissues, deliquescent paraphyses, and asci that generally deliquesce, become detached from the perithecial wall when mature, and have a refractive apical annulus (Voglmayr et al. 2012; Senanayake et al. 2018; Jaklitsch and Voglmayr 2019; Jiang et al. 2019b; Fan et al. 2020; Udayanga et al. 2021). Species of Cryphonectriaceae except *Aurantiosacculuscastaneae* are different from the other diaporthalean taxa by owning orange stromatic tissues at some stage during their life cycle, which turn purple in 3% KOH and yellow in lactic acid (Gryzenhout et al. 2006; Jiang et al. 2019a).

Trees and shrubs of *Elaeocarpus* (Elaeocarpaceae) are evergreen plants, of which several species are planted along streets and in parks. *E.apiculatus* and *E.hainanensis* are commonly used as garden trees in Guangdong Province, however, suffering a serious stem blight disease currently. The present study aims to identify the causal agent based on modern taxonomic approaches and to confirm its pathogenicity.

## ﻿Materials and methods

### ﻿Sample survey, fungal isolation and morphology

**Figure 1.**
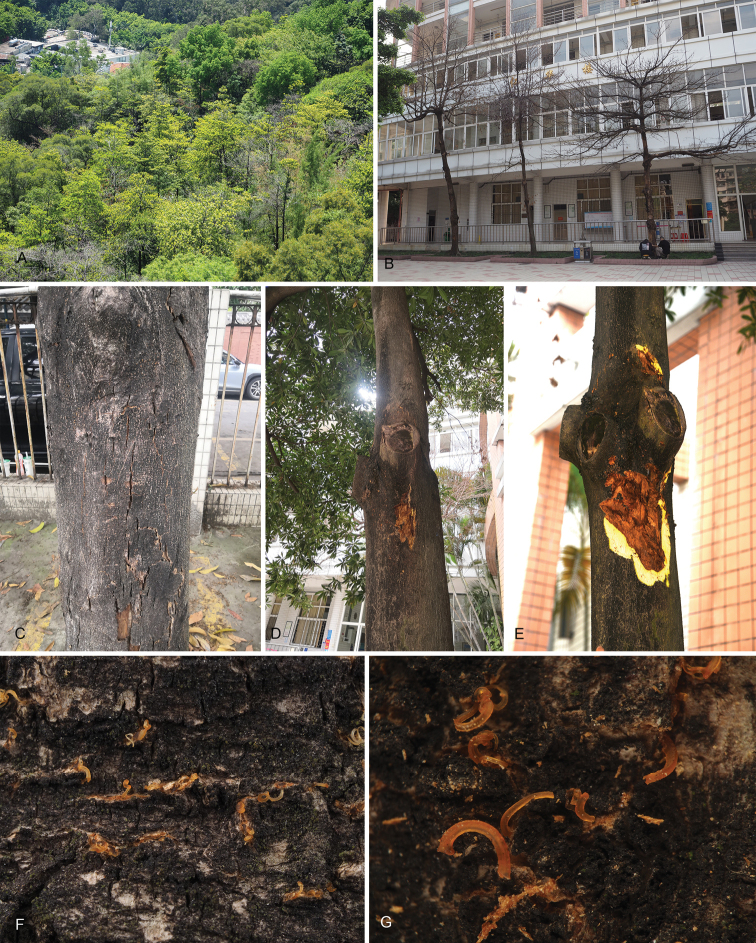
Symptoms caused by *Pseudocryphonectriaelaeocarpicola* on *Elaeocarpus* trees. **A, B** dead trees **C–E** cankered barks **F, G** orange conidial tendrils formed on the cankered barks.

In the present study, we investigated stem blight disease of *Elaeocarpusapiculatus* and *E.hainanensis* in Guangdong Province of China during 2020 and 2022. The disease symptoms on the *Elaeocarpus* trees generally occur on host stems and branches, with cankered barks and orange conidial tendrils (Fig. 1). Most diseased trees died within five months of infection during our investigations. Diseased barks with or without fruiting bodies were collected, packed in paper bags and transferred to the laboratory for isolation.

The diseased barks without orange fungal fruiting bodies were firstly surface-sterilized for 2 min in 75% ethanol, 4 min in 1.25% sodium hypochlorite, and 1 min in 75% ethanol, then rinsed for 2 min in distilled water and blotted on dry sterile filter paper. Then diseased tissues were cut into 0.5 cm × 0.5 cm pieces using a double-edge blade, and transferred onto the surface of potato dextrose agar (PDA; 200 g potatoes, 20 g dextrose, 20 g agar per L), and incubated at 25 °C to obtain pure cultures. The diseased barks with fungal fruiting bodies were checked, and single conidial isolates were obtained from conidiomata by removing the mucoid conidial masses and spreading the suspension onto the surface of PDA. Agar plates were incubated at 25 °C to induce germination of the conidia. After inoculation for up to 48 h, single germinating conidium was then transferred to clean plates under a dissecting stereomicroscope with a sterile needle. The cultures were deposited in China Forestry Culture Collection Center (CFCC, http://cfcc.caf.ac.cn/), and the specimens in the herbarium of the Chinese Academy of Forestry (CAF, http://museum.caf.ac.cn/).

The morphological data of the new taxa in the present study were based on the conidiomata formed on the cankered barks, supplemented by cultural characters. The conidiomata were sectioned and photographed under a dissecting microscope (M205 C, Leica, Wetzlar, Germany). The conidiogenous cells and conidia were immersed in tap water, then the microscopic photographs were captured with an Axio Imager 2 microscope (Zeiss, Oberkochen, Germany) equipped with an Axiocam 506 color camera, using differential interference contrast (DIC) illumination. More than 50 conidia were randomly selected for measurement. Culture characters were recorded from PDA after 7 d incubation at 25 °C in the dark.

### ﻿DNA extraction, PCR amplification and phylogenetic analyses

The fungal genomic DNA was extracted from mycelia grown on cellophane-covered PDA following the method in Doyle and Doyle (1990). DNA was checked by electrophoresis in 1% agarose gel, and the quality and quantity were measured using a NanoDrop 2000 (Thermo Scientific, Waltham, MA, USA). Four partial loci, ITS and LSU regions, *tef1* and *rpb2* genes were amplified by the following primer pairs: ITS1 and ITS4 for ITS (White et al. 1990), LR0R and LR5 for LSU (Vilgalys and Hester 1990), EF1-688F and EF2 for *tef1* (Carbone and Kohn 1999), and RPB2-5F and RPB2-7cR for *rpb2* (Liu et al. 1999). The polymerase chain reaction (PCR) conditions were as follows: an initial denaturation step of 5 min at 94 °C, followed by 35 cycles of 30 s at 94 °C, 50 s at 48 °C (ITS and LSU) or 54 °C (*tub2*) or 55 °C (*rpb2*), and 1 min at 72 °C, and a final elongation step of 10 min at 72 °C. PCR products were assayed via electrophoresis in 2% agarose gels. DNA sequencing was performed using an ABI PRISM 3730XL DNA Analyser with a BigDye Terminator Kit v.3.1 (Invitrogen, Waltham, MA, USA) at the Shanghai Invitrogen Biological Technology Company Limited (Beijing, China).

The sequences obtained in the present study were assembled using SeqMan v. 7.1.0, and reference sequences were retrieved from the National Center for Biotechnology Information (NCBI), based on recent publications (Chen et al. 2018; Jiang et al. 2019a, 2020; Wang et al. 2020). The sequences were aligned using MAFFT v. 6 and corrected manually using MEGA v. 7.0.21 (Katoh and Toh 2010).

The phylogenetic analyses of combined matrixes of the ITS-LSU loci and four loci (ITS-LSU-*tef1*-*rpb2*) were performed using Maximum Likelihood (ML) and Bayesian Inference (BI) methods. ML was implemented on the CIPRES Science Gateway portal (https://www.phylo.org) using RAxML-HPC BlackBox 8.2.10 (Miller et al. 2010; Stamatakis 2014), employing a GTR-GAMMA substitution model with 1000 bootstrap replicates. Bayesian inference was performed using a Markov Chain Monte Carlo (MCMC) algorithm in MrBayes v. 3.0 (Ronquist and Huelsenbeck 2003). Two MCMC chains, starting from random trees for 1000000 generations and trees, were sampled every 100^th^ generation, resulting in a total of 10000 trees. The first 25% of trees were discarded as burn-in of each analysis. Branches with significant Bayesian Posterior Probabilities (BPP > 0.9) were estimated in the remaining 7500 trees. Phylogenetic trees were viewed with FigTree v. 1.3.1 and processed by Adobe Illustrator CS5. The nucleotide sequence data of the new taxon were deposited in GenBank, and the GenBank accession numbers of all accessions included in the phylogenetic analyses are listed in Table 1.

**Table 1.** Isolates and GenBank accession numbers used in the phylogenetic analyses.

Species	Isolate	GenBank Accession Number			
		ITS	LSU	*tef1*	*rpb2*
*Amphilogiagyrosa*	CBS 112922*	AF452111	AY194107	MN271818	MN271782
*Amphilogiagyrosa*	CBS 112923	AF452112	AY194108	MN271819	MN271783
*Aurantioporthecorni*	CMW 10526	DQ120762	AF408343	NA	NA
*Aurantioporthecorni*	CBS 245.90	MN172403	MN172371	MN271822	MN271784
*Aurantiosacculusacutatus*	CBS 132181*	JQ685514	JQ685520	MN271823	NA
*Aurantiosacculuseucalyptorum*	CBS 130826*	JQ685515	JQ685521	MN271824	MN271785
*Aurantiosacculuscastaneae*	CFCC 52456*	MH514025	MH514015	NA	MN271786
*Aurapexpenicillata*	CBS 115740*	AY214311	AY194103	NA	NA
*Aurapexpenicillata*	CBS 115742	AY214313	MN172372	NA	NA
*Aurapexpenicillata*	CBS 115801	MN172404	MN172373	NA	MN271787
*Aurifilummarmelostoma*	CBS 124928*	FJ890495	MH874934	MN271827	MN271788
*Aurifilummarmelostoma*	CBS 124929	FJ882855	HQ171215	MN271828	MN271789
*Capillaureumcaryovora*	CBL02*	MG192094	MG192104	NA	NA
*Celoporthedispersa*	CBS 118782*	DQ267130	HQ730853	HQ730840	NA
*Celoportheeucalypti*	CBS 127190*	HQ730837	HQ730863	HQ730850	MN271790
*Celoportheguangdongensis*	CBS 128341*	HQ730830	HQ730856	HQ730843	NA
*Celoporthesyzygii*	CBS 127218*	HQ730831	HQ730857	HQ730844	NA
*Celoporthewoodiana*	CBS 118785*	DQ267131	MN172375	JQ824071	MN271791
*Chrysomorbuslagerstroemiae*	CBS 142594*	KY929338	KY929328	MN271830	NA
*Chrysomorbuslagerstroemiae*	CBS 142592	KY929330	KY929320	MN271831	NA
*Chrysoportheaustroafricana*	CBS 112916*	AF292041	AY194097	MN271832	NA
*Chrysoportheaustroafricana*	CBS 115843	AF273473	MN172377	MN271833	NA
*Chrysoporthecubensis*	CBS 118654*	DQ368773	MN172378	MN271834	NA
*Chrysoporthecubensis*	CBS 505.63	AY063476	MN172379	MN271835	MN271792
*Chrysoporthehodgesiana*	CBS 115854*	AY692322	MN172380	MN271836	MN271793
*Chrysoporthehodgesiana*	CBS 115744	AY956970	MN172381	MN271837	NA
*Chrysoportheinopina*	CBS 118659*	DQ368777	MN172382	MN271838	NA
*Chrysoporthesyzygiicola*	CBS 124488*	FJ655005	MN172383	MN271839	NA
*Chrysoporthezambiensis*	CBS 124503*	FJ655002	MN172384	MN271840	NA
*Corticimorbussinomyrti*	CBS 140205*	KT167169	KT167179	MN271841	MN271794
*Corticimorbussinomyrti*	CBS 140206	KT167170	KT167180	MN271842	MN271795
*Cryphonectriacitrina*	CBS 109758*	MN172407	EU255074	MN271843	EU219342
*Cryphonectriadecipens*	CBS 129351	EU442657	MN172385	MN271844	MN271796
*Cryphonectriadecipens*	CBS 129353	EU442655	MN172386	MN271845	MN271797
*Cryphonectriajaponica*	CFCC 52148	MH514033	MH514023	MN271846	NA
*Cryphonectriamacrospora*	CBS 109764	EU199182	AF408340	NA	EU220029
*Cryphonectrianeoparasitica*	CFCC 52146*	MH514029	MH514019	MN271847	NA
*Cryphonectriaparasitica*	ATCC 38755	MH843497	MH514021	NA	DQ862017
*Cryphonectriaparasitica*	CFCC 52150	AY141856	EU199123	MN271848	NA
*Cryphonectriaquercus*	CFCC 52138*	MG866024	NA	MN271849	NA
*Cryphonectriaquercicola*	CFCC 52141*	MG866027	NA	MN271850	NA
*Cryphonectriaradicalis*	CBS 112917	AF452113	AY194101	NA	NA
*Cryptometrionaestuescens*	CBS 124007*	GQ369457	MN172387	MN271851	MN271798
*Cryptometrionaestuescens*	CBS 124008	GQ369458	HQ171211	MN271852	MN271799
*Diaportheeres*	LC3198	KP267873	KY011845	KP267947	NA
*Diversimorbusmetrosiderotis*	CBS 132866*	JQ862871	JQ862828	MN271857	NA
*Diversimorbusmetrosiderotis*	CBS 132865	JQ862870	JQ862827	MN271858	NA
*Endothiachinensis*	CFCC 52144*	MH514027	MH514017	MN271860	NA
*Holocryphiaeucalypti*	CBS 115842*	MN172411	MN172391	MN271882	MN271804
*Holocryphiacapensis*	CBS 132870*	JQ862854	JQ862811	MN271883	NA
*Holocryphiagleniana*	CBS 132871*	JQ862834	JQ862791	MN271884	NA
*Holocryphiamzansi*	CBS 132874*	JQ862841	JQ862798	MN271885	NA
*Immersiportheknoxdaviesiana*	CBS 132862*	JQ862765	JQ862755	MN271886	MN271805
*Immersiportheknoxdaviesiana*	CBS 132863	JQ862766	JQ862756	MN271887	MN271806
*Latruncellusaurorae*	CBS 125526*	GU726947	HQ730872	MN271888	NA
*Latruncellusaurorae*	CBS 124904	GU726946	HQ171213	MN271889	NA
*Luteocirrhusshearii*	CBS 130776*	KC197021	KC197019	MN271890	MN271807
*Luteocirrhusshearii*	CBS 130775	KC197024	KC197018	MN271891	MN271808
*Microthiahavanensis*	CBS 115855	DQ368735	MN172393	NA	MN271811
*Microthiahavanensis*	CBS 115841	DQ368736	MN172394	NA	NA
*Microthiahavanensis*	CBS 115758	DQ368737	MN172395	NA	NA
*Myrtonectriamyrtacearum*	CMW 46433*	MG585736	MG585750	NA	NA
*Myrtonectriamyrtacearum*	CMW 46435	MG585737	MG585751	NA	NA
*Parvosmorbuseucalypti*	CSF2060	MN258787	MN258843	MN258829	NA
*Parvosmorbusguangdongensis*	CSF10437	MN258795	MN258851	MN258837	NA
***Pseudocryphonectriaelaeocarpicola***	CFCC 57515*	**ON489048**	**ON489050**	**ON456916**	**ON456918**
***Pseudocryphonectriaelaeocarpicola***	**CFCC 57516**	**ON489049**	**ON489051**	**ON456917**	**ON456919**
*Rostraureumtropicale*	CBS 115725*	AY167435	MN172399	MN271895	MN271814
*Rostraureumtropicale*	CBS 115757	AY167438	MN172400	MN271896	MN271815
*Ursicollumfallax*	CBS 118663*	DQ368755	EF392860	MN271897	MN271816
*Ursicollumfallax*	CBS 118662	DQ368756	MN172401	MN271898	MN271817

Note: NA, not applicable. Ex-type strains are marked with *, and strains from the present study are in black bold.

### ﻿Pathogenicity tests

Three isolates of the new species *Pseudocryphonectriaelaeocarpicola* (ex-type strain: CFCC 57515, CFCC 57516 and CFCC 57517) were used for inoculations, and PDA plugs were used as the negative control. Three isolates were grown on PDA for four days at 25 °C before the tests. Inoculations were performed on 2-year-old seedlings of *Elaeocarpusapiculatus* and *E.hainanensis*, respectively. A total of 40 healthy seedlings were used for the pathogenicity tests. Five seedlings were inoculated with each isolate and the negative control. Inoculations were conducted following the method in Jiang et al. (2019a). The results were evaluated after ten days by measuring the lengths of the lesions on the cambium. The re-isolations were made from the resultant lesions from all tested seedlings by cutting small pieces of discolored xylem and placing them onto the PDA plates. Re-isolates were identified based on the ITS sequences. Differences among isolates in lesion length were analyzed by one-way analysis of variance (ANOVA) followed by least significant difference (LSD) tests. Statistical analysis was carried out by R software (v. 3.4.3) and considered as significant at *p* < 0.05.

## ﻿Results

### ﻿Incidence and isolates

Surveys of *Elaeocarpusapiculatus* and *E.hainanensis* stem blight were conducted in Guangdong Province during 2020 and 2022. Disease incidence was evaluated based on the percentage of the two hosts showing symptoms of all the investigated plants. As shown in Table 2, the disease incidences are all above 85% in seven locations, which indicates this disease poses a serious threat to these two tree hosts.

**Table 2.** Occurrence and incidence of *Elaeocarpusapiculatus* and *E.hainanensis* stem blight in different locations in Guangzhou City.

District	Location	Host	Diseased trees	Dead trees	Healthy Trees	Total	Disease incidence (%)
Tianhe	Longdong Street	*E.apiculatus*	9	10	0	19	100
Tianhe	Guangdong tree Park	*E.apiculatus*	14	9	2	25	92
Tianhe	Shuanglin Street	*E.apiculatus*	18	4	2	24	91.67
Tianhe	Guangdong Eco-Engineering Polytechnic	*E.apiculatus*	11	2	0	13	100
Tianhe	South China Botanical Garden	*E.apiculatus*	5	3	1	9	88.89
Liwan	Meihua Middle School	*E.hainanensis*	3	5	0	8	100
Yuexiu	Luhu Park	*E.apiculatus*	41	21	6	68	91.18

A total of 42 isolates were obtained from the symptomatic tissues of *E.apiculatus* and *E.hainanensis*, and six isolates from the conidiomata formed on the cankered barks. They are identical based on the sequence data, hence isolates CFCC 57515 from *E.hainanensis* and CFCC 57516 from *E.apiculatus* were selected for phylogenetic analyses.

### ﻿Phylogenetic analyses

**Figure 2.**
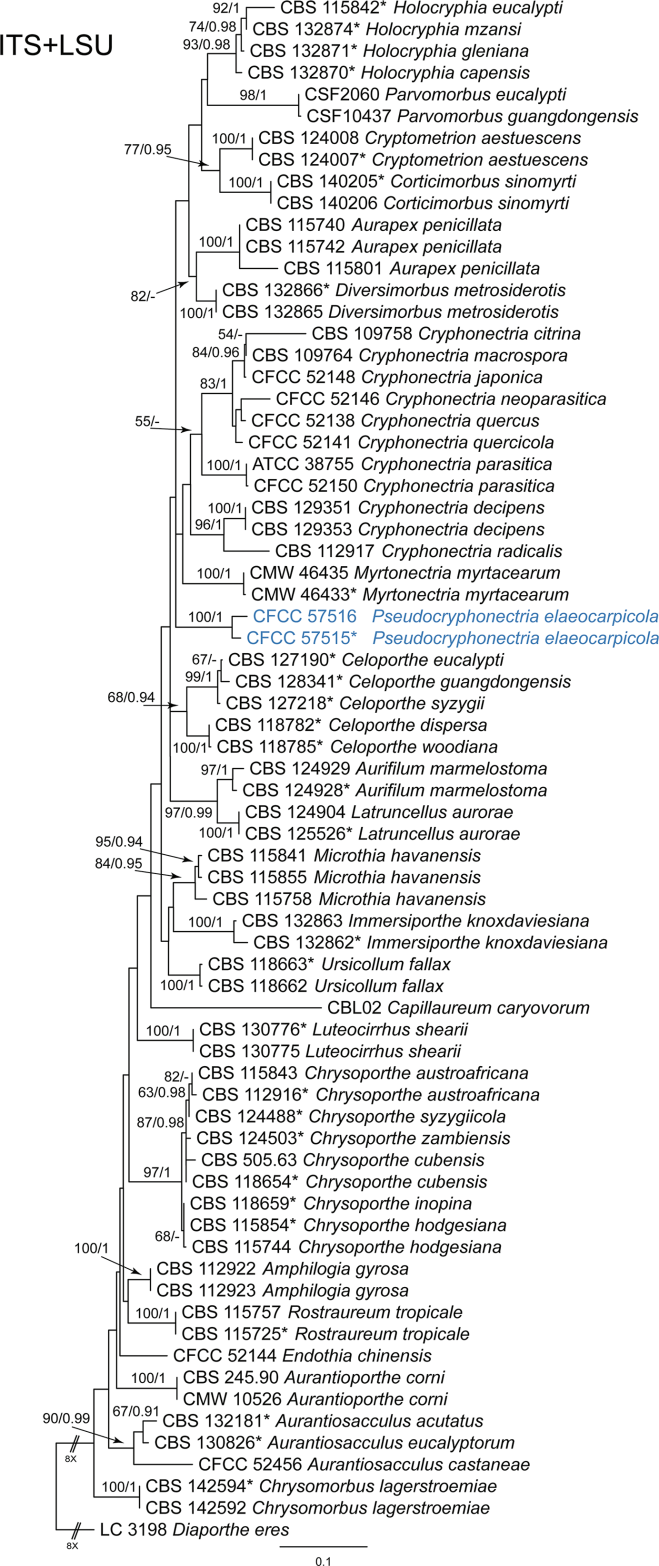
Phylogram of Cryphonectriaceae resulting from a maximum likelihood analysis based on combined ITS and LSU loci. Numbers above the branches indicate ML bootstrap values (left, ML-BS ≥ 50%) and Bayesian Posterior Probabilities (right, BPP ≥ 0.9). The tree is rooted with *Diaportheeres* (LC 3198). Isolates from the present study are marked in blue, and ex-type strains are marked with *.

The sequence dataset of the ITS-LSU gene matrix was analysed to infer the genus and species relationships within Cryphonectriaceae. The dataset consisted of 71 sequences including one outgroup taxon, *Diaportheeres* (LC 3198). A total of 1580 characters including gaps were included in the phylogenetic analysis. The topologies resulting from ML and BI analyses of the concatenated dataset were congruent (Fig. 2). Isolates from the present study formed a distinct clade from the other genera of Cryphonectriaceae, which represents an undescribed genus.

**Figure 3.**
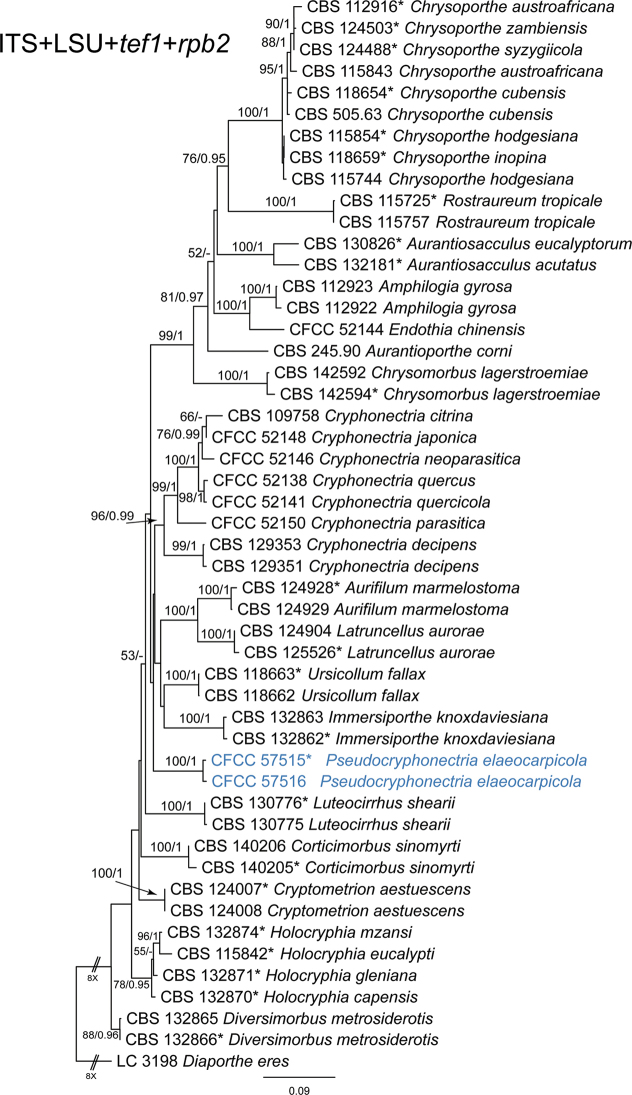
Phylogram of Cryphonectriaceae resulting from a maximum likelihood analysis based on combined ITS, LSU, *tef1* and *rpb2* loci. Numbers above the branches indicate ML bootstrap values (left, ML-BS ≥ 50%) and Bayesian Posterior Probabilities (right, BPP ≥ 0.9). The tree is rooted with *Diaportheeres* (LC 3198). Isolates from the present study are marked in blue, and ex-type strains are marked with *.

The combined four-loci sequence dataset (ITS, LSU, *tef1* and *rpb2*) was further analysed to compare with results of the phylogenetic analyses of the ITS-LSU gene matrix. The dataset consisted of 50 sequences including one outgroup taxon, *Diaportheeres* (LC 3198). A total of 3226 characters including gaps (726 for ITS, 854 for LSU, 811 for *tef1* and 835 for *rpb2*) were included in the phylogenetic analysis. The topologies resulting from ML and BI analyses of the concatenated combined dataset were congruent (Fig. 3). Isolates from the present study formed a distinct clade which was congruent with that shown in Fig. 2.

### ﻿Taxonomy

#### ﻿ Pseudocryphonectria

Taxon classificationFungiDiaporthalesCryphonectriaceae

Huayi Huang gen. nov.

794B2492-3F60-58B2-9645-A0B7C49D8EB5

 844044

##### Etymology.

Named derived from *pseudo*- and the genus name *Cryphonectria*.

##### Type species.

*Pseudocryphonectriaelaeocarpicola* Huayi Huang

##### Description.

Sexual morph: Unknown. Asexual morph: *Conidiomata* pycnidial, aggregated or solitary, immersed under the host bark, subglobose to pulvinate, yellow to orange, multilocular, single ostiolate, forming long orange tendrils. *Conidiophores* cylindrical, aseptate, hyaline, sometimes reduced to conidiogenous cells. *Conidiogenous cells* lining inner cavity of conidiomata, phialidic, ampulliform, with attenuated or truncate apices, hyaline, smooth. *Conidia* dimorphic. *Microconidia* minute, aseptate, hyaline, smooth, cylindrical, straight. *Macroconidia* aseptate, hyaline, smooth, obclavate, straight or slightly curved.

##### Notes.

*Pseudocryphonectria* has typical orange cryphonectriaceous stromata, which turns purple the 3% KOH and yellow in lactic acid. This genus is characterized by its dimorphic conidia from the same conidioma, which is different from the other genera of Cryphonectriaceae (Chen et al. 2013, 2016, 2018; Beier et al. 2015; Jiang et al. 2020).

#### ﻿ Pseudocryphonectria elaeocarpicola

Taxon classificationFungiDiaporthalesCryphonectriaceae

Huayi Huang sp. nov.

13EA0C08-31E7-579D-9475-B2CEF57EF13A

 844045

Figs 4 , 5

##### Etymology.

Named after the host genus, *Elaeocarpus*.

##### Description.

Sexual morph: Unknown. Asexual morph: *Conidiomata* pycnidial, aggregated or solitary, immersed under the host bark, subglobose to pulvinate, yellow to orange, 500–1200 μm wide, 150–450 μm high, multilocular, single ostiolate, forming long orange tendrils. ***Conidiophores*** cylindrical, aseptate, hyaline, sometimes reduced to conidiogenous cells. ***Conidiogenous cells*** lining inner cavity of conidiomata, phialidic, ampulliform, with attenuated or truncate apices, hyaline, smooth, 12.8–25.7 × 1.7–3.2 μm (n = 50). ***Conidia*** dimorphic. ***Microconidia*** minute, aseptate, hyaline, smooth, cylindrical, straight, (3.1–)3.3–4(–4.4) × (1.5–)1.6–2(–2.1) μm (n = 50), L/W = 1.6–2.7. ***Macroconidia*** aseptate, hyaline, smooth, obclavate, straight or slightly curved, (4.6–)5.1–6.1(–6.6) × (1.4–)1.6–2(–2.2) μm (n = 50), L/W = 2.5–3.9.

**Figure 4.**
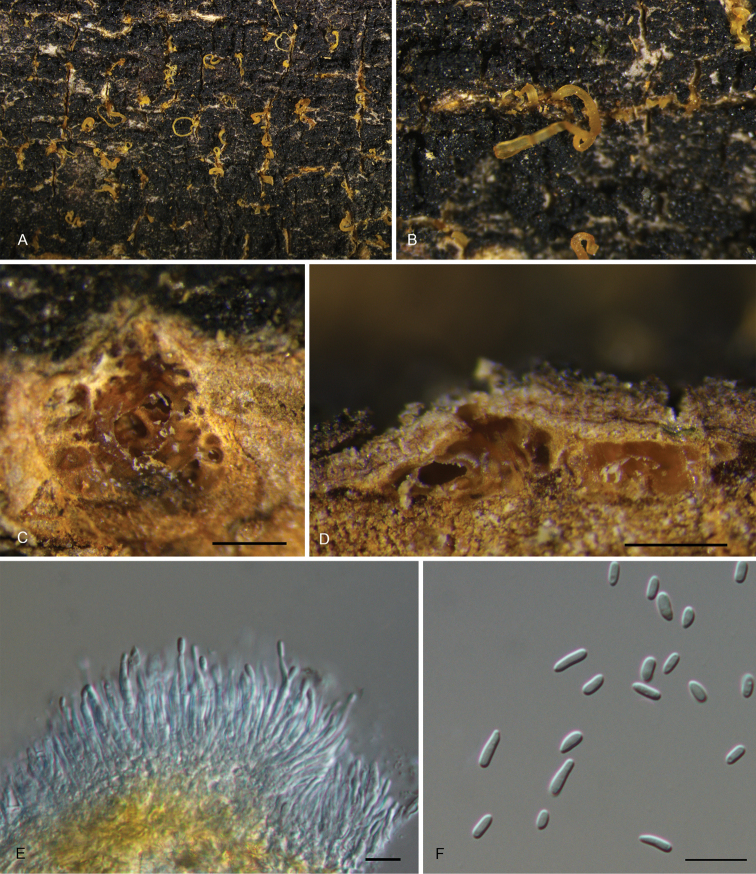
Morphology of *Pseudocryphonectriaelaeocarpicola* from *Elaeocarpushainanensis***A, B** habit of conidiomata on the host stem **C** transverse section through the conidioma **D** longitudinal section through the conidioma **E** conidiogenous cells giving rise to conidia **F** macroconidia and microconidia. Scale bars: 300 μm (**C, D**); 10 μm (**E, F**).

##### Culture characters.

***Colonies*** on PDA flat, spreading, with aerial mycelium and entire margin, white to mouse grey, forming abundant orange conidiomata with orange conidial masses.

**Figure 5.**
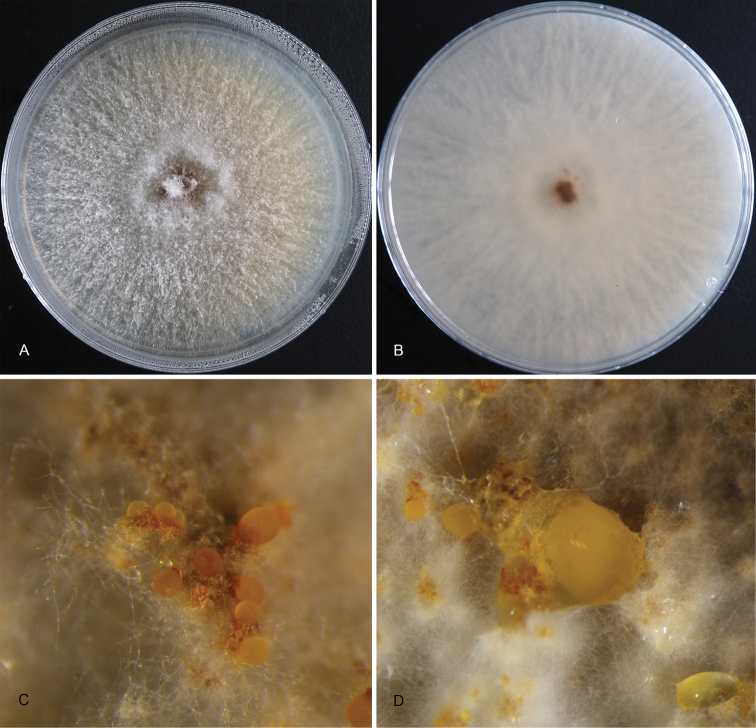
Morphology of *Pseudocryphonectriaelaeocarpicola* from PDA**A, B** colonies **C, D** orange conidiomata.

##### Specimens examined.

China, Guangdong Province, Guangzhou City, Meihua middle school, 23°8'37.94"N, 113°14'18.12"E, 24 m asl, on stems and branches of *Elaeocarpushainanensis*, 7 March 2022, Huayi Huang (CAF800051 holotype; ex-type living culture, CFCC 57515). Guangdong Province, Guangzhou City, Luhu Park, 23°9'11.15"N, 113°16'46.01"E, 92 m asl, on stems and branches of *E.apiculatus*, Huayi Huang, 15 March 2022 (CAF800055 paratype; ex-paratype living culture, CFCC 57516). Guangdong Province, Guangzhou City, Longdong straight street, 23°11'41.02"N, 113°22'8.33"E, 46 m asl, on stems and branches of *E.apiculatus*, Huayi Huang, 1 April 2022 (DY03, culture, CFCC 57517). Guangdong Province, Guangzhou City, South China botanical garden, 23°11'3.5"N, 113°21'41.53"E, 39 m asl, on stems and branches of *E.apiculatus*, Huayi Huang, 11 April 2022 (DY24, culture, DY24-2). Guangdong Province, Guangzhou City, Linke 1^st^ street, 23°11'35.81"N, 113°22'46.69"E, 74 m asl, on stems and branches of *E.apiculatus*, Huayi Huang, 15 April 2022 (DY32; culture, DY32-1). Guangdong Province, Guangzhou City, Nonglin middle street, 23°11'23.84"N, 113°22'43.08"E, 46 m asl, on stems and branches of *E.apiculatus*, Huayi Huang, 15 April 2022 (DY42, culture, DY42-1).

##### Notes.

*Pseudocryphonectriaelaeocarpicola* is the sole species within the new genus, which causes serious stem blight of *Elaeocarpus* trees. Another notorious pathogen in Cryphonectriaceae, *Cryphonectriaparasitica*, causes serious chestnut worldwide. Morphologically, *P.elaeocarpicola* is similar to *C.parasitica* in the appearance of conidiomata with orange conidial tendrils formed on the host bark. However, *P.elaeocarpicola* can be distinguished from *C.parasitica* by its obvious dimorphic conidia (Jiang et al. 2019a). Phylogenetically, isolates of *P.elaeocarpicola* clustered into a distinct clade in the phylograms of Cryphonectriaceae (Figs 2, 3).

### ﻿Pathogenicity tests

**Figure 6.**
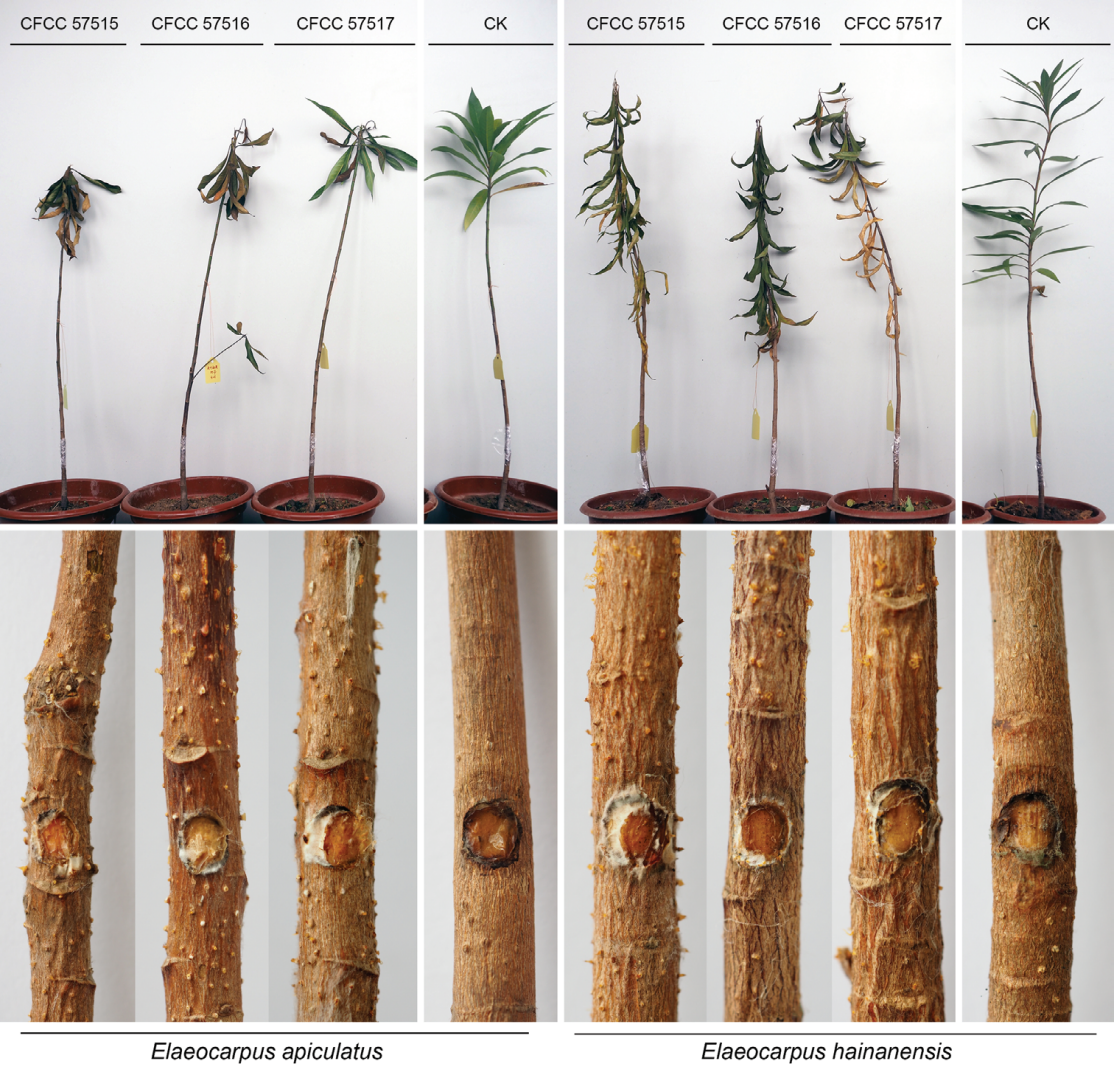
Results of pathogenicity tests on *Elaeocarpusapiculatus* and *E.hainanensis* using isolates CFCC 57515, CFCC 57516 and CFCC 57517. Row 1: appearance of the hosts after incubation in 10 days; row 2: conidiomata formed on the barks.

**Figure 7.**
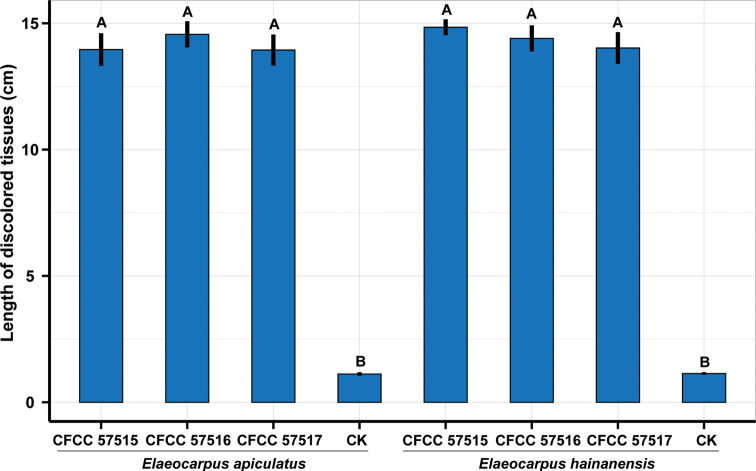
Histogram of lesion lengths resulting from inoculation on *Elaeocarpusapiculatus* and *E.hainanensis* using isolates CFCC 57515, CFCC 57516 and CFCC 57517. Different letters above the error bars indicate treatments that were significantly different (*p* = 0.05).

Ten days after inoculation on young seedlings of *Elaeocarpusapiculatus* and *E.hainanensis*, isolates CFCC 57515, CFCC 57516 and CFCC 57517 all caused death of the host, and formed orange conidiomata on the barks, and the negative control only produced minor lesions (Fig. 6). Statistical analyses of data showed no significant difference among three tested isolates on two hosts of *E.apiculatus* and *E.hainanensis*, however, significantly different from the negative control (Fig. 7). Isolates were obtained from lesions produced on tested seedlings, and were identical to *Pseudocryphonectriaelaeocarpicola* based on the sequence data and morphology of conidiomata formed on the barks. Hence, *P.elaeocarpicola* can quickly infect *E.apiculatus* and *E.hainanensis*, and kill the hosts.

## ﻿Discussion

In the present study, the causal agent of stem blight on *Elaeocarpusapiculatus* and *E.hainanensis* was identified using both morphological and phylogenetical approaches, which revealed a new genus and species, namely *Pseudocryphonectriaelaeocarpicola*. Further pathogenicity test conducted on the two original hosts *E.apiculatus* and *E.hainanensis* confirmed the high virulence of the fungal pathogen. In ten days, the fungus can infect the host and kill both *E.apiculatus* and *E.hainanensis*. As shown in Table 2, the pathogen kills more than a half of the diseased adult trees during our investigations, which is similar to its relative fungus *Cryphonectriaparasitica* in pathogenicity (Rigling and Prospero 2017). Luckily, we timely discovered the fungus and report it herein, and the disease control studies have been in progress.

In the fungal order Diaporthales, many species were reported as forest pathogens causing leaf spots, cankers, fruit rot or blight diseases (Visentin et al. 2012; Pasche et al. 2016; Shuttleworth and Guest 2017; Jiang et al. 2021b; Pan et al. 2021; Lin et al. 2022), moreover, cryphonectriaceous members are known to be serious pathogens (Chen et al. 2013, 2016; Beier et al. 2015; Ferreira et al. 2019; Wang et al. 2020). This family is easily recognized based on the disease symptoms and their obvious orange conidioma formed on the cankered barks, together with their hyaline and small conidia (Gryzenhout et al. 2006). However, within this family, genera are similar in morphology which are usually distinguished by the molecular data (Jiang et al. 2020; Wang et al. 2020). Most genera in this family are known to own only one or two species; this may be caused by most samples on important trees like Fagaceae, Melastomataceae, and Myrtaceae and limited samples from the other hosts (Jiang et al. 2020; Wang et al. 2020). In the present study, *Elaeocarpus* (Elaeocarpaceae) usually being overlooked hosts, were found to be new hosts of Cryphonectriaceae pathogens.

There is still room for further exploration, such as the infection opportunity, sources of the primary infection and the alternative hosts of the pathogen. More importantly, the effective control methods to protect *Elaeocarpus* hosts are urgent to be studied due to the quick infection and high virulence.

## Supplementary Material

XML Treatment for Pseudocryphonectria

XML Treatment for Pseudocryphonectria elaeocarpicola
